# Toll-Like Receptor Ligand Based Adjuvant, PorB, Increases Antigen Deposition on Germinal Center Follicular Dendritic Cells While Enhancing the Follicular Dendritic Cells Network

**DOI:** 10.3389/fimmu.2020.01254

**Published:** 2020-06-19

**Authors:** Christina Lisk, Rachel Yuen, Jeff Kuniholm, Danielle Antos, Michael L. Reiser, Lee M. Wetzler

**Affiliations:** ^1^Section of Infectious Diseases, Department of Medicine, Boston Medical Center, Boston, MA, United States; ^2^Department of Microbiology, Boston University School of Medicine, Boston, MA, United States; ^3^Department of Microbiology and Immunology, University of Pittsburgh School of Medicine, Pittsburgh, PA, United States; ^4^DRK-Blutspendedienst, BaWü-Hessen gGmbH, Frankfurt, Germany

**Keywords:** adjuvants, TLR-ligand based adjuvants, PorB, neisseria, TLR2, follicular dendritic cells, dendritic cells, antigen deposition

## Abstract

Vaccines are arguably one of the greatest advancements in modern medicine. Subunit vaccines comprise the majority of current preparations and consist of two main components—antigen and adjuvant. The antigen is a small molecule against which the vaccine induces an immune response to provide protection via the immunostimulatory ability of the adjuvant. Our laboratory has investigated the adjuvant properties of Toll-like receptor (TLR) ligand-based adjuvants, especially the outer membrane protein from *Neisseria mengingitidis*, PorB. In this current study we used PorB, along with CpG, an intracellular TLR9 agonist, and a non-TLR adjuvant, aluminum salts (Alum), to further investigate cellular mechanisms of adjuvanticity, focusing on the fate of intact antigen in the germinal center and association with follicular dendritic cells (FDCs). FDCs are located in the B cell light zone of the germinal center and are imperative for affinity maturation. They are stromal cells that retain whole intact antigen allowing recognition by the B cell receptor of the germinal center B cells. Our studies demonstrate that TLR ligands, but not Alum, increase the FDC network, while PorB and Alum increased colocalization of FDC and the model soluble antigen, ovalbumin (OVA). As PorB is the only adjuvant tested that induces both a higher number of FDCs and increased deposition of antigen on FDCs, it has the greatest ability to increase FDC-antigen interaction, essential for induction of B cell affinity maturation. These studies demonstrate a further mechanism and potential superiority of PorB as an adjuvant and its influence on antibody production.

## Introduction

Vaccines are one of the most significant advancements in modern medicine (1–5). Utilizing vaccines, smallpox has been eradicated and measles infection rate dropped by 80% from 2000 to 2017 (6). Yet there are still infectious diseases where the empirical methods have failed to produce a successful vaccine (7–10). In order to produce more effective vaccines, researchers have developed subunit vaccines, which consist of two main components—antigen and adjuvant (11–14). The antigen is a small molecule against which a protective response can be induced, but only with the addition of the adjuvant, which provides immunostimulation to induce this response (15–17). Antigen alone is usually unable to sufficiently provide protection therefore the addition of adjuvants has become critical. Adjuvants were described by Charles Janeway as the immunologist “dirty little secret” which defined pathogen associated molecular patterns (PAMPs) from microbial origins (18). PAMPS are recognized as “non-self” molecules by pattern recognition receptors (PRRs) on innate immune cells (19). There are multiple families of PRRs including membrane-bound receptors and cytoplasmic receptors. One subclass of PRRs are Toll-like receptors (TLRs). TLRs can be extracellularly located or within endosomes (20–22). TLR engagement activates downstream intracellular signaling cascades and induce cellular activation, activation marker expression and cytokine and chemokine production (19, 23–32). These characteristics account for the fact that many TLR-ligands are effective vaccine adjuvants (21, 33–38). Investigators can select certain TLR-ligand based adjuvants to examine specific cellular pathways within the immune system and draw conclusions based on protection and adaptive immune responses (36, 39–43).

Our laboratory has investigated the adjuvant properties of the major outer membrane protein from *Neisseria mengingitidis*, PorB. PorB is a TLR2/1 ligand, and is able to significantly increase co-stimulatory ligand expression and cytokine production in antigen presenting cells (APC) (44). In addition, PorB can increase antigen loaded APC trafficking to the lymph node (45), induce germinal center formation (46), and enhance antigen specific antibody production, CD4^+^ T cell activation (47), and cross presentation allowing for CD8^+^ T cell activation (45). We have mainly used subcutaneous immunizations for these studies; however, the effect of adjuvants of the microenvironment of the draining lymph nodes from these injections has not be extensively investigated. In the current studies, utilizing fluorochrome labeled antigen, we investigated the fate of intact antigen in the draining lymph nodes 24 h post-immunization in mice and whether adjuvants influence this process. In addition to PorB, we have also examined the effect of CpG, a TLR-9 agonist used as an intracellular TLR-ligand based adjuvant, and a non-TLR adjuvant, aluminum salts (Alum). Both of these adjuvants have been shown to increase cytokine expression in innate immune cells (48), and increase antigen specific antibodies (49). To date, the exact cellular interaction from immune cells to illicit a protective response after vaccination including adjuvants have not been fully described.

Multiple cellular interactions are needed to induce a protective antibody response. One critical initial step is antigen reaching the lymph node, either by trafficking as processed antigen in dendritic cells (DCs) or as free intact antigen from the lymphatic vessels. DCs are the primary APC during vaccine induced immune responses, taking up antigen at the immunization site, processing such antigen while trafficking to the secondary lymphoid organs (SLO) (50). The antigen containing DCs are needed to stimulate T follicular helper cells (Tfh), which can then further enhance antigen specific B cell activation during the germinal center response (51). Free intact antigen exits lymphatic drainage via subcapsular marginal zone macrophages and are eventually deposited on follicular dendritic cells (FDCs), likely by a non-cognate B, though this is unclear (52). FDCs are stromal cells within the lymph nodes and spleen which are located in the B cell light zone of the germinal center and are vital for induction of B cell somatic hypermutation and antibody (Ab) affinity maturation. They could also be involved with B cell differentiation into memory B cells or long-lived plasma cells (53). FDCs recycle antigen and antigen-antibody complex (known as immune complexes, IC) to the cell surface via actin-requiring processes (54) without proteolytically processing the antigen. Once the B cell receptor is engaged with the native antigen on the FDC, cytokines and chemokines are secreted for induction of B cell survival, allowing for: (1) exiting of the germinal center completely if high affinity interactions with intact antigen occur, (2) re-entering the B cell dark zone of the germinal center if moderate affinity to intact antigen occurs, for further activation by antigen specific Tfhs along with induction of somatic hypermutation, or (3) apoptosis if they have low affinity for their antigen (55). To date, very few studies have investigated how adjuvants influence this process, especially in regards to antigen association with FDCs (56, 57).

The studies presented here were designed to determine the effect of adjuvants on the initial steps involved in induction of B cell activation in the germinal center, which would subsequently lead to induction of high affinity antibodies. We examined the effect of adjuvants on the level of intact antigen present in the lymph node, deposition of this antigen on FDCs and the overall quality of the FDC network. These studies highlight the manner by which adjuvants, especially PorB, may influence desired vaccine antigen interaction with cells in the germinal center to influence antibody production essential for vaccine efficacy. We have published multiple papers describing PorB's adjuvant characteristic which resulted in higher antigen specific antibody levels as well as more diverse antigen specific subtypes than other adjuvants tested (44, 46) which substantiates our approach taken in these studies.

## Methods

### Animals

Four to eight-week-old female and male C57Bl/6J (referred to as “wild type,” stock #000664) mice were obtained from Jackson Laboratories (Bar Harbor, ME). All mice were maintained within the Laboratory Animals Science Center (LASC) at Boston University School of Medicine. The Boston University Institutional Animal Care and Use Committee (IACUC) approved all research conducted using animal models (protocol number 201800024). All experiments involving the mice were performed in accordance within the relevant guidelines and regulations as defined by our IACUC.

### Murine Immunizations

Groups of mice received one of the following immunization preparations: ovalbumin (OVA) fluorescently labeled with Alexa 594 (OVA-A594) alone (Life technologies), OVA-A594 + PorB, OVA-A594 + CpG (Invitrogen, Cat#ODN1826), or OVA-A594 + Alum (Aluminum hydroxide, Sigma, Cat#A8222). OVA was used at 10 μg per mouse, PorB and CpG at 10 μg per mouse and Alum at 200 μg per mouse based on previous publications (44, 46). An initial kinetic study using OVA-A594 given alone or with PorB, as above, was performed to determine the optimal time point for lymph node isolation to examine effects of adjuvants on antigen deposition on FDCs (Supplemental Figure 1A). All mice were injected subcutaneously near the base of the tail. Draining lymph nodes were isolated after euthanasia 24, 48, or 72 h after immunization (69). The nodes were embedded in optimal cutting temperature (OCT) medium (Richard Allan Scientific, Kalamazoo, MI, USA) in molds and used for immunohistochemistry.

### Immunohistochemistry

Draining iliac and inguinal lymph nodes were isolated 24 h after immunization (46) and put into molds containing OCT medium and frozen on dry ice. Tissues were sectioned on a Microm HM 550 (Microm International GmBH, Germany). Eight micrometer sections were obtained and placed on Colorfrost Plus slides and stored at −80°C until staining. Sections were air dried for 15 min at room temperature, fixed in acetone at −20°C for 10 min, and air dried for 10 min. Sections were re-hydrated in TBS buffer with 0.05% Tween-20 (TBS-T) then blocked for 1 h at room temperature with TBS-T with 5% BSA. Sections were rinsed with PBS and then stained with conjugated (CD11c, Biolegend, Cat#117309) and primary (FDC-M1, BD Biosciences, Cat#551320) antibodies overnight at 4°C followed by three rinses with PBS. Secondary antibody (anti-rat 488, Biolegend, Cat#405418) was added to the slides for 1 h at room temperature followed by three washed in PBS. Antibody concentration for the primary was 1:100. Conjugated and secondary was used at 1:200 dilution. Stained sections were mounted in Fluoroshield mounting medium with DAPI (Abcam), dried overnight, and sealed with clear nail polish. A Leica SP5 confocal microscope (Leica AG) was used to examine the sections using the Leica LAS AF software using the 10x (HC PL FLUORTAR 10.0X0.3 Dry) and 63x oil immersion objectives. All images were captured with 4 lines average at 200 Hz. The images were arranged and analyzed using FIJI/ImageJ (NIH).

### Image Analysis

After images were obtained from the Lecia SP5 and imported into FIJI/ImageJ (NIH). The background was subtracted for each image separately using a rolling ball radius of 20.0 pixels. Mean fluorescence intensity (MFI) was determined using the ImageJ and the measurement tool. Colocalization between DCs or FDCs and OVA was determined using the JaCoP plugin in ImageJ calculating the Pearson Colocalization Coefficient (Supplemental Figures 1B,C). To determine the MFI of OVA associated with DCs and FDCs, Mander's correlation coefficient was determined from the JaCoP plugin as a percentage of OVA (58) (Supplemental Figure 2) and then multiplied by the total MFI of OVA within the lymph node.

### Flow Cytometry of Follicular Dendritic Cells and Dendritic Cells

Single cell suspensions were created from inguinal lymph nodes 24 h post injection. Briefly, lymph nodes were placed in cold PBS and were manually minced on a petri dish with a scalpel. The samples were transferred to a 24-well plate **(**Fisher Scientific, Cat #08-772-1H**)**, incubated with DMEM containing 2% FBS (ThermoFisher, Cat#26140079), 33.3 mg/ml collagenase type IV (ThermoFisher, Cat#17104019), and 2,500 U/mL DNase I (ThermoFisher, Cat#18047019). Samples were incubated for 1 h at 37°C. After which, the samples were strained through 70 μm filter. Cells were incubated with a live/dead stain (Biolegend, Cat#423105) for 30 min, in the dark at 4°C. Cells were then washed with 5x FACS Buffer (PBS, 0.5%BSA, and 2% EDTA) and spun down. Cells were then incubated with CD16/CD32 Fc block (eBioscience, 48-0032-82) for 10 min in the dark at room temperature. Cells were then plated in a 96 V-well bottom plate (Corning, CLS3896-48EA) and stained. All dilutions were 1:200. Antibodies included: CD19-BUV395 (BD Horizon, 563557), CD3—eFlour (Invitrogen, 48-0032-82), CD11c—APC (BD Pharmigen, 550261). Cells were the analyzed on an LSRII. The gating strategy is shown in Supplemental Figures 3A,B. Animals were vaccinated with OVA lacking the Alexa594 fluorochrome as negative controls as shown in Supplemental Figure 3C. Single cell suspensions for FDCs were performed similarly. The samples were strained through 70 μm filter, although not pushed through to ensure the integrity of the FDCs remained intact. Samples were then stained for live/dead, Fc block, and conjugated antibodies. All antibody dilutions were 1:200 unless otherwise noted. CD21/CD35—BV421, CD45—APC, CD19—BUV395 (1:400), ICAM-1—FITC. Gating strategy is shown in Supplemental Figure 4A. A fluorescence minus one (FMO) was stained for all colors within the panel excluding CD21/CD35 shown in Supplemental Figure 4B. All samples were analyzed on an LSRII, a machine available within the Boston University flow core, on a low flow setting.

### Statistics

Statistics were calculated in GraphPad Prism (version 8.0). Pearson Correlation Coefficients were analyzed as described above. ANOVA with Sidak's multiple comparisons test was used for all other analysis. ns, not significant, ^*^p < 0.05, ^**^p < 0.01, ^***^p < 0.001, ^****^p < 0.0001

## Results

### Mean Fluorescence Intensity of OVA Increased With PorB Injections

**Figure 1:**
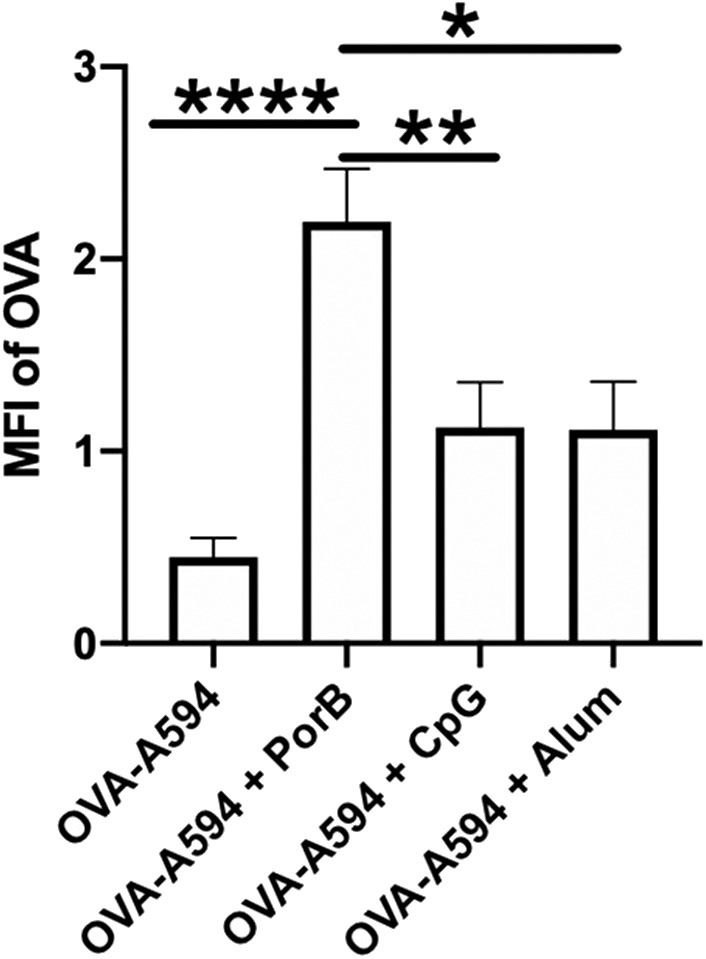
PorB increases antigen presence in draining lymph node. Mean fluorescent intensity (MFI) of antigen (OVA) in draining lymph nodes 24 h post vaccination of either OVA-A594, OVA-A594 + PorB, OVA-A594 + CpG, or OVA-A594 + Alum. MFI of OVA was quantified by the ImageJ measurement tool after the subtraction of the background. Representative of three experiments. *n* = 5–7, *p < 0.05, **p < 0.01, ****p < 0.001.

At first, we needed to confirm that adjuvants could influence the presence of intact antigen in the SLO. This outcome is essential because antigen presence within the lymph node is a primary factor contributing to the establishment of an adaptive immune response. The groups we analyzed consisted of mice immunized with OVA labeled with Alexa594 (OVA-A594), OVA-A594 + PorB, OVA-A594 + CpG, and OVA-A594 + Alum. To determine whether adjuvants influenced antigen presence within the lymph nodes of the animals in this study, the average MFI of OVA in the draining lymph nodes was calculated from immunohistochemistry (IHC) images (Figure 1). Interestingly, only PorB appeared to increase the amount of labeled OVA within the lymph nodes. This increase was significant over other adjuvants used in these studies.

### Follicular Dendritic Cell Networks Are Increased by TLR-Ligand Based Adjuvants

**Figure 2:**
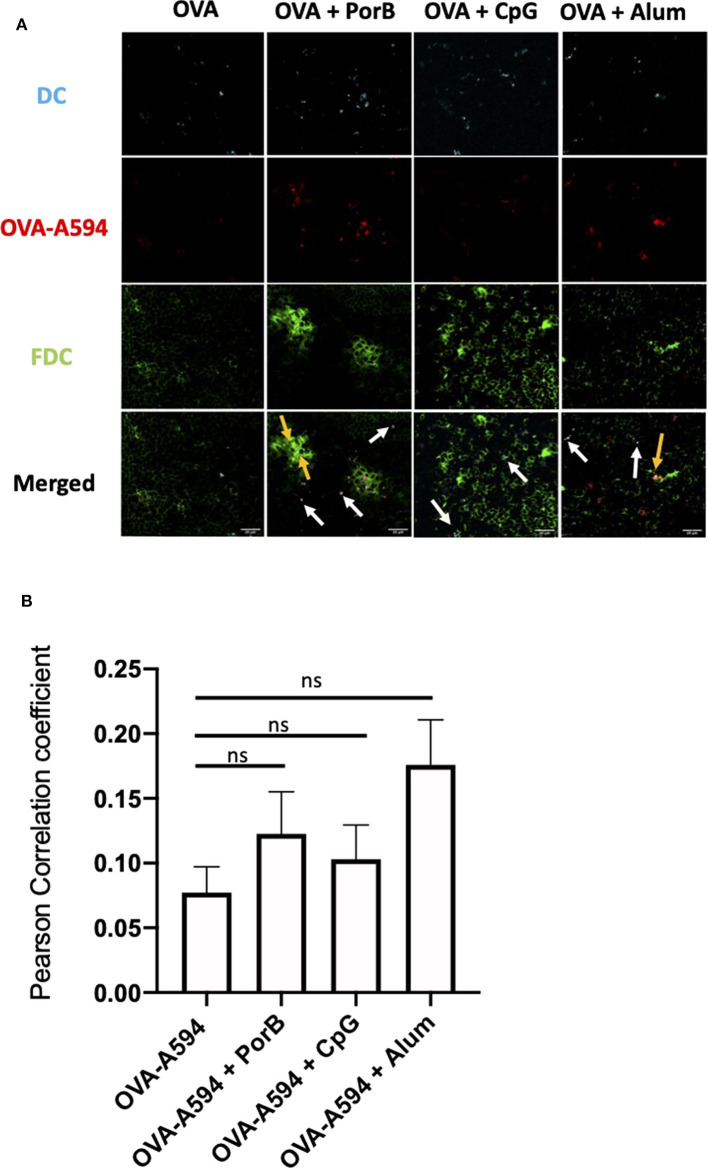
Evaluation of the effect of Adjuvants on FDC Networks. **(A)** Representative images for FDC expression in draining lymph nodes 24 h post subcutaneous injections of either OVA-A594, OVA-A594 + PorB, OVA-A594 + CpG, or OVA-A594 + Alum. FDC expression is shown as a heat map where white indicates the highest signal to pixel ratio and blue sows the lowest signal to pixel ratio. Scale bar is 100 μM. One of 3 representative experiments is shown. **(B)** Mean fluorescence intensity (MFI) quantification from ImageJ of FDC networks in draining lymph nodes 24 h post injection of either OVA-A594, OVA-A594 + PorB, OVA-A594 + CpG, or OVA-A594 + Alum. Multiple FDC networks were measured within individual lymph nodes. *n* = 9/group. *p < 0.05, ***p < 0.001 **(C)** Frequency of FDC in draining lymph nodes 24 h post subcutaneous injections with OVA-A594 ± adjuvants. Gating strategy is shown in Supplemental Figure 4. **(D)** Cell counts of FDC in draining lymph nodes 24 h post subcutaneous injections with OVA-A594 ± adjuvants. **(E)** MFI of intercellular adhesion molecule 1 (ICAM-1) from FDC gate in draining lymph nodes 24 h post subcutaneous injections with OVA-A594 ± adjuvants. **(F)** MFI of complement receptors 1 and 2 (CR1/2) from FDC gate in draining lymph nodes 24 h post subcutaneous injections with OVA-A594 ± adjuvants. *n* = 9 per group *p < 0.05, **p < 0.01; ns, not significant.

To determine if the adjuvants directly affected the quality of the FDC networks, we performed immunofluorescent staining on draining lymph nodes from immunized mice using primary antibody FDC-M1, which is the common marker for FDCs, and an Alexa 488 secondary antibody. This study included mice immunized with four different preparation as previously described: OVA-A594, OVA-A594 + PorB, OVA-A594 + CpG, and OVA-A594 + Alum. Samples were analyzed by ImageJ to calculate the MFI values for FDC-M1. Figure 2A displays representative images of FDC-M1 labeling in draining lymph nodes 24 h post immunization as a heat map, where white indicates the highest signal to pixel ratio and blue shows the lowest signal to pixel ratio. Lymph node FDC-M1 labeling was low in mice immunized with OVA alone or Alum + OVA. However, it was greatly increased when TLR-ligand based adjuvants (PorB and CpG) were used as shown in both Figures 2A,B.

To confirm these results, flow cytometry was utilized to quantify FDC numbers in the draining lymph nodes. The gating strategy is shown in Supplemental Figure 4A. FDCs were defined as CD19^−^CD45^−^Cr1/Cr2^+^ICAM-1^+^. Fluorescence minus one (FMO) was used to ensure the cells isolated were Cr1/Cr2^+^ (Supplemental Figure 4B). As shown in Figures 2C,D, the flow cytometry data matched the IHC data both in frequency and cell counts of FDC. Animals vaccinated with PorB or CpG with OVA-A594 demonstrated a significant increase in FDC numbers in the draining lymph nodes as compared to the use of Alum + OVA-A594 or OVA-A594 alone. To further confirm that the increase in FDCs were not just due to measuring an increase in expression of activation markers intercellular adhesion molecule 1 (ICAM1) or complement receptors 1 and 2 (CR1/2), mean fluorescent intensity was calculated via FlowJo. As shown in Figure 2 *Immunology* E and F, no significant differences were measured for ICAM1 or CR1/2. These results, in addition to the IHC measurements, led us to concluded that TLR-ligand based adjuvants, PorB and CpG, significantly increased FDC numbers within the germinal centers of draining lymph node 24 h post subcutaneous injection.

### Antigen Deposition on Follicular Dendritic Cells Is Increased With PorB and Alum

**Figure 3:**
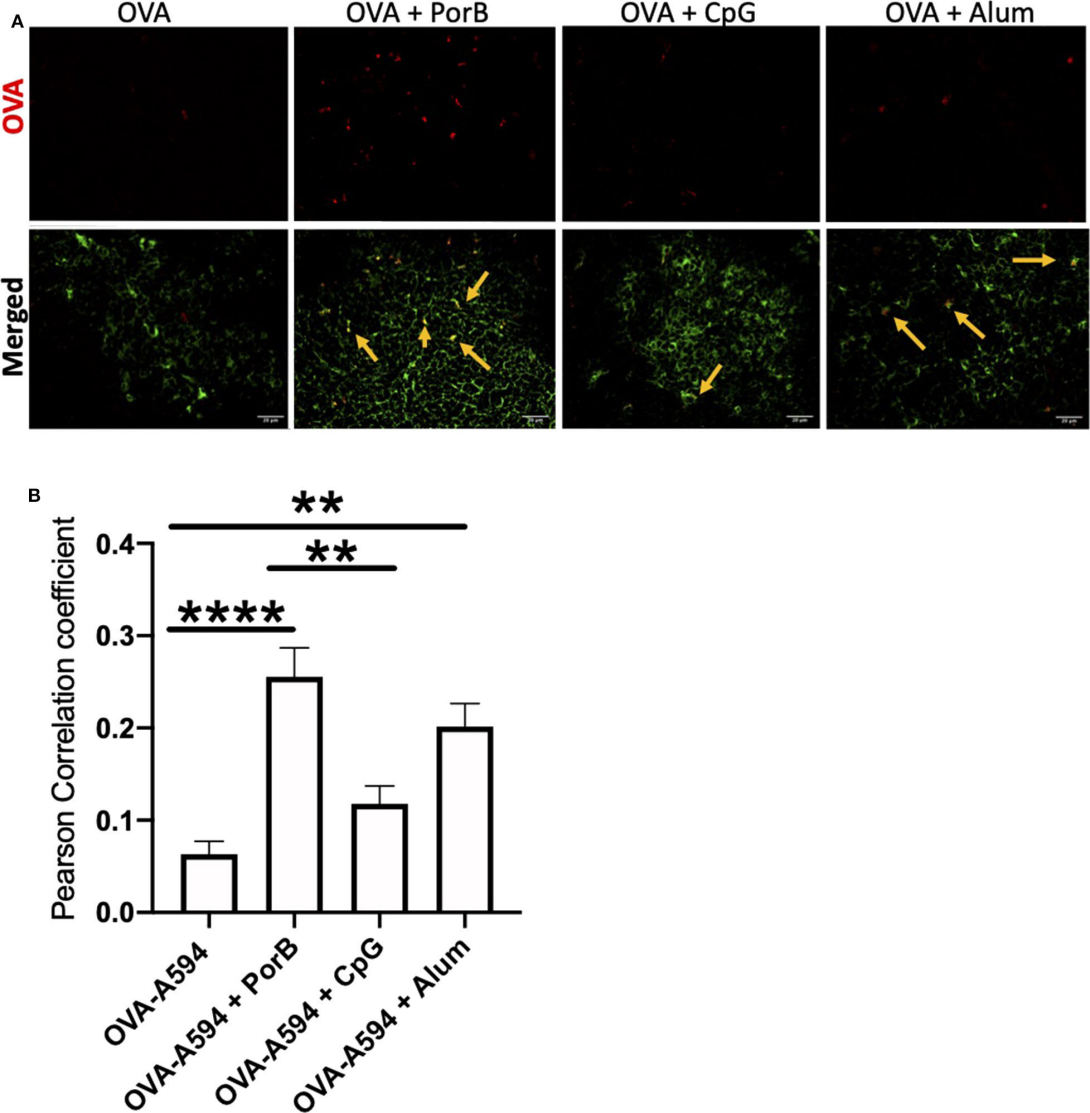
Adjuvants effect colocalization of antigen onto FDCs. **(A)** Representative IHC images from draining lymph nodes from B6 control mice 24 h post subcutaneous injections. FDCs are shown in green. OVA-594, used as a non-immunogenic antigen, is shown in red. Areas of colocalization are shown with yellow arrows. Scale bar represents 20 μM. One out of 3 representative experiments is shown. Images were taken at 63x objective using a Leica SP5 microscope. **(B)** Quantification of Colocalization of fluorescently labeled OVA-A594 with FDCs within draining lymph nodes 24 h post subcutaneous injections. Colocalization was assessed using Pearson Correlation coefficients calculated with JaCoP plugin in ImageJ after background subtraction. *n* = 9–12, **p < 0.01, ****p < 0.001.

As antigen deposition on FDCs is important and more biologically relevant than FDC numbers, the ability of adjuvants to influence antigen deposition onto FDCs was examined. Draining lymph nodes from immunized mice described above were examined by immunofluorescence microscopy to determine colocalization of labeled OVA with FDCs. As displayed in Figure 3A, non-adjuvanted OVA-A594 was minimally present in the lymph node 24 h post-immunization. When adjuvants were included, OVA-A594 was detectable within the lymph node, regardless of the adjuvant administered. Lymph nodes from mice given OVA-A594 + PorB had the most OVA present. Lymph nodes from mice given OVA-A594 + PorB or OVA-A594 + Alum had multiple areas of colocalization of OVA with FDC (shown by yellow arrows). There appeared to be less colocalization when CpG was used. JaCoP was used to quantify colocalization between OVA and FDC signal in each tissue section, as previously performed in our lab (45). The Pearson Correlation coefficient from JaCoP confirmed significant increases of colocalization in the lymph nodes from mice given OVA-A594 + PorB and OVA-A594 + Alum as compared to lymph nodes from mice given OVA-A594 alone (Figure 3B).

### FDC Colocalization Is Independent of Antigen Loaded Dendritic Cells

**Figure 4:**
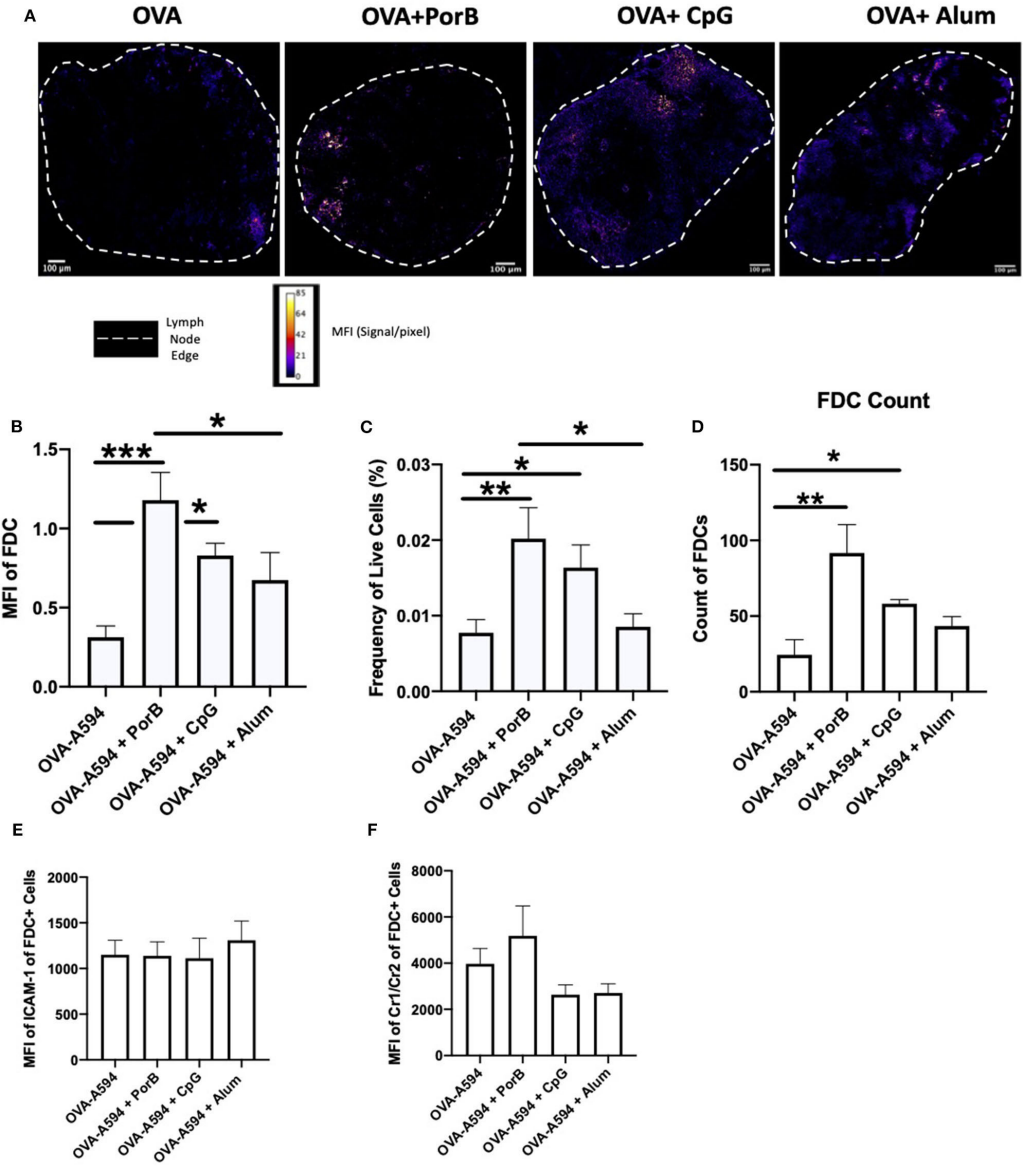
FDC antigen deposition is independent of antigen loaded DCs. Representative IHC images of draining lymph nodes from mice 24 h post subcutaneous injections are shown in **(A)** where FDC is shown in green, OVA-594, used as a non-immunogenic antigen, is shown in red and dendritic cells (DCs, CD11c) are shown in cyan. Areas of colocalization between DC and OVA are shown with white arrows. Areas of colocalization between FDC and OVA are shown with yellow arrows. Images were taken at 63x objective using a Leica SP5 microscope. One out of 2 representative experiments is shown. **(B)** Pearson's correlation coefficient between FDC and DC (CD11c) in draining lymph nodes 24 h post subcutaneous injections. Colocalization was assessed using Pearson Correlation coefficients calculated with JaCoP plugin in ImageJ after subtracting the background and using an unsharp mask filter. *n* = 7/group. **(C)** Frequency of live FDCs from the draining lymph node 24 h after injections quantified by flow cytometry. **(D)** FDC count from the draining lymph node 24 h after injections quantified by flow cytometry. **(E)** MFI of ICAM-1 within the FDC+ gating strategy from the draining lymph node 24 h after injections. **(F)** MFI of Cr1/Cr2 within the FDC+ gating strategy from the draining lymph node 24 h after injections. *p < 0.05, **p < 0.01, ***p < 0.0001.

To ensure the OVA correlation with FDCs was not due to concomitant presence antigen loaded DCs, we first examined draining lymph nodes by IHC for DCs (as labeled by anti-CD11c fluorochrome labeled Ab) along with the FDCs staining to determine if the two colocalized. Figure 4A demonstrates that a majority of the OVA colocalized with either DCs or FDCs in all treatment groups. The white arrows emphasize areas of colocalization between DCs and OVA, whereas the yellow arrow illustrates FDCs colocalization with OVA. JaCoP was used to determine whether OVA colocalization with FDCs vs. DCs were uniquely and separate. As shown in Figure 4B, the Pearson Correlation coefficient was not significant for any of the adjuvants tested for induction of direct association between DCs and FDCs. These data emphasize that the OVA deposition on FDCs is increased with adjuvants when compared to OVA alone and, in general, is independent of CD11c^+^ DCs trafficking OVA within the lymph node.

### PorB Significantly Increases OVA Association With FDCs and DCs as Compared to CpG or Alum

**Figure 5:**
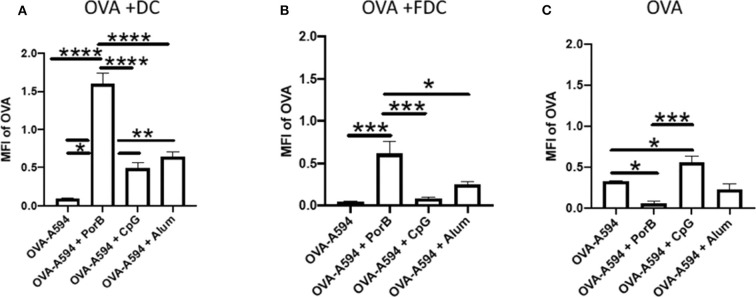
OVA-A594 distribution between DC, FDC, and unassociated OVA within draining lymph node 24 h post subcutaneous injection. MFI of OVA associated with DC **(A)**, FDC **(B)**, or unassociated OVA **(C)** was assessed using the JaCoP plugin within ImageJ and determining the Mander's correlation coefficient after subtracting the background. The correlation coefficient is the percentage of DCs or FDCs associated with OVA. This percentage was then multiplied by the total MFI of OVA within the lymph node (Figure 1) to determine MFI of OVA associated with either DCs or FDCs. These data were assessed in draining lymph nodes 24 h post injection of either OVA-A594, OVA-A594 + PorB, OVA-A594 + CpG, or OVA-A594 + Alum. *n* = 5–7 *p < 0.05, **p < 0.01, ***p < 0.001, ****p < 0.0001.

We next determined how the increase of antigen within the SLO was distributed between antigen loaded DCs, antigen deposition onto FDC, or unassociated with either of these cell types. Mander's correlation coefficients (JaCop within ImageJ) were used to determine the percentage of OVA that was associated with either DCs, FDCs, or neither. This correlation coefficient allows for spilt channels of correlation to be determined (58). The percentages of OVA correlated with either DCs or FDCs were then multiplied by the MFI of OVA (Figure 1) to determine the MFI of OVA associated with DCs, FDCs, or neither. All adjuvants had a significant increase in MFI of OVA associated with DCs, but PorB's increase was significantly greater than the other adjuvants tested (Figure 5A), which is consistent with our previous data (45). OVA association with FDCs was also significantly increased when PorB was used, as compared to the other adjuvants (Figure 5B). Lastly, levels of “unassociated OVA,” which we defined as the remaining percentage of OVA that was not associated with either DCs or FDCs (Unassociated OVA = 1-[Mander's coefficient for OVA/DC + Mander's coefficient for OVA/FDC]) was determined. Figure 5C shows that OVA-A594 + PorB had the only significant decrease in unassociated OVA. Interestingly, OVA-A594 + CpG had a significant increase in unassociated OVA. These data emphasize that the PorB, as an adjuvant, induced a significant increase in antigen associated with both DCs and FDCs when compared to other adjuvants.

### Dendritic Cells Numbers in Draining Lymph Node Were Increased With the Use of Adjuvants

**Figure 6:**
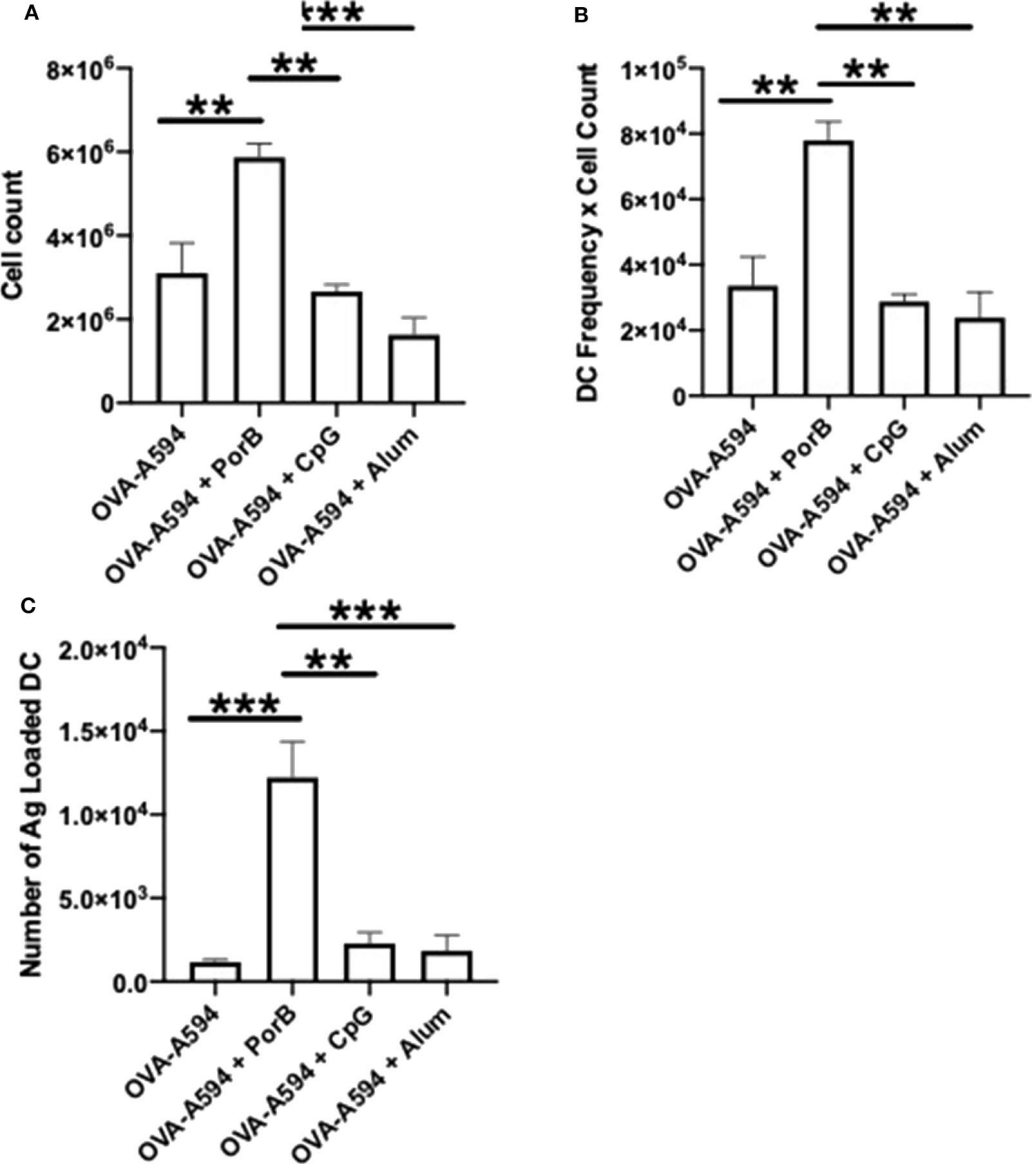
PorB increases cell count, DCs, and antigen loaded DCs. **(A)** Single cell suspensions were used to calculate cell counts within the draining lymph node 24 h post injection of either OVA-A594, OVA-A594 + PorB, OVA-A594 + CpG, or OVA-A594 + Alum. **(B)** DCs from draining lymph nodes of animals 24 h post injection from either OVA-A594, OVA-A594 + PorB, OVA-A594 + CpG, or OVA-A594 + Alum. Gating strategy is shown in Supplemental Figure 3A. Statistics were calculated by ordinary one-way ANOVA with Sidak's multiple comparisons test. *p < 0.05, **p < 0.01 **(C)** Antigen loaded DCs from draining lymph nodes of animals 24 h post injection from either OVA-A594, OVA-A594 + PorB, OVA-A594 + CpG, or OVA-A594 + Alum. Gating strategy is shown in Supplemental Figures 3B,C. Statistics were calculated by ordinary one-way ANOVA with Sidak's multiple comparisons test. *n* = 3, data represents one of 3 experiments. *p < 0.05, **p < 0.01, ***p < 0.0001.

DCs are a critical APC for the adaptive immune responses. To determine if adjuvants influence the number of DCs present in within draining lymph nodes after immunization, single cell suspensions of these lymph nodes were obtained 24 h post injection with OVA-A594, OVA-A594 + PorB, OVA-A594 + CpG, or OVA-A594 + Alum. We have previously examined this parameter for PorB (45) but have never compared this to other adjuvants, the gating strategy is shown in Supplemental Figure 3A. Our analysis showed a significant increase in cell count with PorB adjuvanted vaccines as well as a significant increase in DCs within the draining lymph node (Figures 6A,B). Interestingly, and supporting our previous work, PorB vaccinations showed a significant increase in antigen loaded DCs 24 h post subcutaneous injections (Figure 6C).

## Discussion

In order to understand the influence of different adjuvants on multiple immune response related pathways within the lymph node, we utilized the following vaccine adjuvants: PorB, a TLR1/2 ligand-based adjuvant, well-studied in our lab (59), along with CpG, a TLR9 agonist that has been previously used as an adjuvant, and Alum, a TLR-independent adjuvant. PorB, CpG and Alum have all been shown to increase antigen-specific antibody responses by our group and (46, 60–65). OVA-A594 was used as our model antigen based on previous studies in our laboratory as, on its own, does not induce innate immune activation or adaptive immune responses. Moreover, previous studies utilized tools and reagents unique for OVA, including defined T cell epitopes, MHC tetramers and TCR transgenic mice that recognize these epitopes (45, 46). Initially, we demonstrated that PorB was able to significantly increase the presence of OVA within the draining lymph nodes 24 h post subcutaneous immunization as compared to CpG or Alum (Figure 1). The specificity of the adaptive immune response is dependent on the presence of intact antigen on FDCs in the lymph node and processed antigen trafficked by DCs to the lymph node (54).

FDCs are critical for antibody production by providing intact antigen to B cell receptors (BCR) (55, 66). Depending on the affinity of the BCR, the B cell will either leave the germinal center, return to the dark zone for further somatic hypermutation, or become apoptotic based on cytokine expression from the FDCs (55). Here-in we investigated how adjuvants may affect these cells, especially in regards to antigen deposition. We demonstrated that TLR-ligand based adjuvants, PorB and CpG, both significantly increased the FDC network 24 h after a subcutaneous immunization by both confocal microscopic and flow cytometric analyses without significant increases in overall MFI of either ICAM-1 or CR1/2 within the FDC gating strategy (Figure 2). FDCs are stromal cells within the lymph node (67, 68); the increase is likely due to an overall increase in cellularity induced by the adjuvants (45). An increase in FDCs would allow for more surface area onto which more intact antigen can be deposited during the initiation of the adaptive immune response. This could lead to greater interaction with B cells and increased B cell receptor specificity by allowing for more B cells to come in contact with the deposited antigen, and increasing the kinetics of developing high affinity BCRs and antibodies. Interestingly, the use of PorB or Alum as adjuvants with OVA, significantly increased antigen deposition on the FDCs, as opposed to the use of CpG (Figure 3). However, Alum did not increase the number of FDCs overall; PorB was the only adjuvant studied that both increased FDC number and intact antigen deposition. Other studies have focused on passive immune complex (IC) injections to show deposition onto FDCs (52). While these passive studies are important, antigen still needs to be deposited on the FDCs to allow for BCR interactions allowing for B cell somatic hypermutation and enhanced Ab affinity. Our studies uniquely focused on primary exposures to antigen and the deposition onto FDCs.

The effect of PorB and the other adjuvants on DC trafficking to the lymph nodes and antigen association with DCs was also analyzed. DCs are a critical APCs for the adaptive immune response (50). Usage of PorB or Alum as an adjuvant increased the numbers DCs within the draining lymph nodes (Figure 6B). More importantly, however, only PorB demonstrated significant increases of antigen loaded DCs in these same draining lymph nodes (Figure 6C) (45). The effect of PorB and other adjuvants on DC antigen uptake and trafficking to the lymph nodes is a crucial step for antigen specificity during the adaptive immune response as DCs are the primary cell to present antigen to T cells in the SLO and subsequent activation of antigen specific B cells (50).

The effect of adjuvants on OVA distribution in the lymphoid follicle and germinal center demonstrates, for the first time, the ability of PorB to more efficiently direct intact antigen toward cellular pathways directly involved in antibody production as compared to other adjuvants tested. Figure 5 demonstrates that immunizations including PorB as an adjuvant resulted in the majority of labeled OVA MFI in the lymphoid follicle and germinal center to be associated with either FDCs or DCs (CD11c). As stated, both of these cell types are imperative for effective antibody production due to FDCs interactions with B cells and DC interactions with Tfh cells. Interestingly, PorB was also the only adjuvant to show a significant decrease in OVA MFI that was not associated with either cell type. This emphasizes that PorB has a more targeted effect toward the adaptive immune responses than other adjuvants investigated here.

Overall, these studies emphasize the role adjuvants have on specific cellular mechanisms involved in vaccine induced antibody production. For the first time, follicular dendritic cells were demonstrated to be increased in numbers by both extracellular TLR1/2 agonist, PorB, and intracellular TLR9 agonist, CpG. Of these two, only PorB also increased antigen deposition onto the FDCs. The timepoint for these analyzes were chosen to investigate early innate responses that are involved in the initiation of adaptive immune responses. Yuen and Kuniholm have recently highlighted how PorB has major influences on the immune system as part of its highly effective adjuvant effect such as increasing costimulatory molecule expression on and cytokine production in dendritic cell, s as well as increasing production antigen-specific IgG antibodies including IgG1, IgG2b, and IgG3 subtypes (59). In the studies described above, we have further shown that PorB is able to increase draining lymph node antigen levels, FDC numbers, and increase antigen deposition on these FDCs. This is certainly related to its significant ability to enhance antigen specific antibody production (44). Moreover, consistent with previous data (45), PorB increases trafficking of antigen loaded DCs to the lymph node, separate from the intact antigen deposition on the FDCs. This will allow for both increased B cell affinity maturation and increased antigen specific T cell induction and activation. Together, these data emphasize the pivotal role of adjuvants in immune processes leading to antibody production, along with evidence that PorB has characteristics that may make it a superior adjuvant as compared to others.

## Data Availability Statement

All datasets generated for this study are included in the article/Supplementary Material.

## Ethics Statement

The animal study was reviewed and approved by Boston University IACUC.

## Author Contributions

The work was performed by CL, with help and advice form RY, JK, and MR and technical help from DA. All the work was planned with LW and CL. LW wrote and edited the manuscript. All authors contributed to the article and approved the submitted version.

## Conflict of Interest

The authors declare that the research was conducted in the absence of any commercial or financial relationships that could be construed as a potential conflict of interest.
