# Supplementary material for: Toll-Like Receptor Ligand Based Adjuvant, PorB, Increases Antigen Deposition on Germinal Center Follicular Dendritic Cells While Enhancing the Follicular Dendritic Cells Network
Source: Front Immunol. 2020 Jun 19;11:1254. doi: 10.3389/fimmu.2020.01254 (PMC7318107; doi:10.3389/fimmu.2020.01254)
Supplement: Supplementary file 1 [file Data_Sheet_1.docx]

Supplemental Figure 1: (a) **Experimental design for antigen trafficking within the lymph nodes**. Mice were divided into 3 groups – 72 hours, 48 hours, or 24 hours for post injection euthanasia. These groups were further divided into OVA-A594 or OVA-A594 + PorB. All injections were subcutaneous. n = 4/group. (b) **Flow Chart for Pearson correlation coefficient analysis.** Images were imported into ImageJ and then changed to a 16 bit image. The plugin JaCoP was then utilized to determine Pearson correlation coefficient which were then calculated for statistical significance in PRISM 8.0 using ordinary one-way ANOVA. (c) **Pearson correlation coefficient between OVA and FDC in iliac lymph nodes**. Mice were divided into 3 groups – 72 hours, 48 hours, or 24 hours for post injection euthanasia. These groups were further divided into OVA-A594 or OVA-A594 + PorB. All injections were subcutaneous. *p<0.05, **p<0.01

c.

a.

b.

Supplemental Figure 2: **Pearson correlation coefficient analysis.** Images were imported into ImageJ and then changed to a 16-bit image. The plugin JaCoP was then utilized to determine Manders correlation coefficient. Manders correlation coefficients were then analyzed in Prism 8.0 for significance.

Supplemental Figure 3: **Flow Cytometric Gating Strategies** **for DCs** (a) Gating strategy for DCs in draining lymph nodes. (b) Gating strategy for antigen loaded DCs. Gates were created to remove doublets and dead cells. DCs were then selected from CD3- and CD19- gates. OVA-A594+ gate was created to determine % of DCs with antigen. (c) Fluorescent minus one gate for 594^+^CD11c^+^ events. An animal injected with PBS is shown in pink. An animal injected with OVA-A594 is shown in cyan. These graphs are taken from live CD3^-^CD19^-^ cells.

c.

b.

a.

Supplemental Figure 4: **Flow Cytometric** **Gating Strategies for FDCs** (a) gating strategy for follicular dendritic cells (FDC). (b) Fluorescence minus one (FMO) for Cr1/Cr2. Two samples were analyzed based on the gating strategy in Supplemental figure 2b. One sample contained Cr1/Cr2 antibody (pictured on the left). The other did not (pictured on the right). These are the results of the flow cytometry

a.

b.
